# Introducing extended consultations for patients with severe mental illness in general practice: Results from the SOFIA feasibility study

**DOI:** 10.1186/s12875-023-02152-z

**Published:** 2023-10-05

**Authors:** A. B. R. Jønsson, F. H. J. Martiny, M. K. Søndergaard, J. B. Brodersen, T. D. Due, M. H. Nielsen, C. Bakkedal, J. E. Bardram, K. Bissenbakker, I. Christensen, K. Doherty, P. Kjellberg, S. W. Mercer, S. Reventlow, M. P. Rozing, A. Møller

**Affiliations:** 1 The Section of General Practice and Research Unit for General Practice, Department of Public Health, University of Copenhagen, Copenhagen, Denmark; 2 Center for Health and Society, Department of People and Technology, Roskilde University, Roskilde, Denmark; 3 Department of Community Medicine, The Arctic University of Norway, Tromsø, Norway; 4 Center for Social Medicine, Bispebjerg and Frederiksberg Hospital, Copenhagen, Denmark; 5 The Research Unit for General Practice, University of Southern Denmark, Odense, Denmark; 6 Primary Health Care, Region Zealand, Denmark; 7 Department of Health Technology, Technical University of Denmark, Kongens Lyngby, Denmark; 8 The Danish Centre for Social Science Research, Copenhagen, Denmark; 9 Usher Institute, College of Medicine and Veterinary Medicine, University of Edinburgh, Edinburgh, Denmark

**Keywords:** Primary care, General practice, Severe mental illness, Extended consultations, Qualitative methods, Patient-centred care, Feasibility studies

## Abstract

**Background:**

People with a severe mental illness (SMI) have shorter life expectancy and poorer quality of life compared to the general population. Most years lost are due to cardiovascular disease, respiratory disease, and various types of cancer. We co-designed an intervention to mitigate this health problem with key stakeholders in the area, which centred on an extended consultations for people with SMI in general practice. This study aimed to1) investigate general practitioners’ (GPs) experience of the feasibility of introducing extended consultations for patients with SMI, 2) assess the clinical content of extended consultations and how these were experienced by patients, and 3) investigate the feasibility of identification, eligibility screening, and recruitment of patients with SMI.

**Methods:**

The study was a one-armed feasibility study. We planned that seven general practices in northern Denmark would introduce extended consultations with their patients with SMI for 6 months. Patients with SMI were identified using practice medical records and screened for eligibility by the patients’ GP. Data were collected using case report forms filled out by practice personnel and via qualitative methods, including observations of consultations, individual semi-structured interviews, a focus group with GPs, and informal conversations with patients and general practice staff.

**Results:**

Five general practices employing seven GPs participated in the study, which was terminated 3 ½ month ahead of schedule due to the COVID-19 pandemic. General practices attempted to contact 57 patients with SMI. Of these, 38 patients (67%) attended an extended consultation, which led to changes in the somatic health care plan for 82% of patients. Conduct of the extended consultations varied between GPs and diverged from the intended conduct. Nonetheless, GPs found the extended consultations feasible and, in most cases, beneficial for the patient group. In interviews, most patients recounted the extended consultation as beneficial.

**Discussion:**

Our findings suggest that it is feasible to introduce extended consultations for patients with SMI in general practice, which were also found to be well-suited for eliciting patients’ values and preferences. Larger studies with a longer follow-up period could help to assess the long-term effects and the best implementation strategies of these consultations.

**Supplementary Information:**

The online version contains supplementary material available at 10.1186/s12875-023-02152-z.

## Introduction

People with a severe mental illness (SMI), defined as psychotic disorders, bipolar disorders, and severe degree of depressive disorder, have a 10 to 20-year shorter life expectancy than the general population [1–7]. Most years lost are due to cardiovascular disease, respiratory disease, and various types of cancer [4, 8–11].

Several interventions have tried to address this disparity on multiple levels, i.e. interventions addressing individual, institutional, and socio-environmental risk factors [12], yet they have proved unsuccessful [12, 13]. Contrary to former interventions in the area, the SOFIA project ascribes a pivotal role to primary healthcare in improving the somatic health and quality of life of people with SMI [14]. This project is distinct from earlier attempts to develop interventions for this population group due to its effort in co-designing the intervention with patients, caretakers, and general practitioners (GPs) [15].

The SOFIA project began in 2017 with a 2-year co-design phase that co-developed the intervention in collaboration with relevant stakeholders aiming to align its design with the needs and challenges of patients, general practice, and other stakeholders [15]. The following themes became apparent from the co-design phase: GPs were concerned with the lack of time allocated for addressing what mattered to patients with SMI, and they needed tools to strengthen the patient-doctor relationship with this particular patient group (Jønsson ABR, Svanholm SL, Brodersen JB, Reventlow S, Brostrøm M: General practitioners’ experiences of providing somatic care for patients with severe mental illness: a qualitative study, forthcoming). Further, patients with SMI felt they were not always taken seriously when presenting somatic symptoms in general practice. Often, they were not physically examined and felt misunderstood by health care professionals [16].

### Developing the preliminary SOFIA intervention

Based on findings from the co-design phase, the SOFIA project group developed a preliminary intervention that centred on GPs providing an extended consultation for patients with SMI (up to 45 min). Additionally, GPs received information on relevant collaborative care initiatives and an introductory meeting on conducting the extended consultation using the SOFIA scheme [14] (Table 1).

**Table 1:** The SOFIA scheme

**Welcome**
The patient and general practitioner (GP) agree on the aim of the consultation. Information about the study and participation is repeated. It is orally confirmed that informed consent for study participation has been given.
**SOcial clinical space: The “patient part” of the consultation**
This opening part of the consultation aims to establish a positive relationship between the patient and the GP. The patient has the opportunity to present his or her complaints and through clarifying the patient’s thoughts, feelings, and notions regarding these complaints, the GP sets an agenda for the consultation. Suggestions for open questions the GP could ask are: “How are you? Is there anything that you would like to focus on today? Are there any other concerns that I should be aware of? Is there anything in particular that you hope to gain from today’s meeting and is there anything that you hope that I can help you with?” Dependent on the study arm the patient is allocated to, results from the surveys about the quality of life may be discussed. The GP is instructed to probe for areas that need attention and needs that should be focused on, especially if the patient’s sum score on any of the six scales indicates poor quality of life in the construct measured by the scale. The GPs are instructed to ask whether the patient experiences suicidal thoughts (if so general practitioners are instructed to follow the SOFIA handbooks’ guide on talking about suicide). If not already known, GPs ask about possible substance abuse and self-harm (if yes, see the SOFIA handbook for referrals).
***FI*nd any symptoms for undiagnosed or undertreated somatic diseases: The “GPs’ part” of the consultation**
The middle section of the consultation aims to collect information on current diagnoses and their treatments and to detect possible, unrecognized, and undertreated disorders or overdiagnosed and/or overtreated conditions. The GPs are instructed to ask about known diseases and current treatments and any symptoms that the patient may experience. The GPs will perform a focused physical somatic diagnostic interview, based on any somatic concerns that the patient and GP agree upon. The patient must be physically examined, even if they have no physical complaints, because of the delayed and altered bodily experience often accompanying SMI. The GPs conclude this part of the consultation with a brief review of current medication and, if relevant, make a plan to optimize pharmacological treatment. The GPs discuss adherence challenges related to treatment, possible side effects, and any possible considerations or wishes for medication changes with the patient. If required, a pharmacologist can be consulted by email. If required, a follow-up consultation focusing on medications will be scheduled.
***A*gree on individual care plan (final step of the SOFIA scheme)**
During the final part of the consultation, an individual care plan is made. The GP and the patient will discuss current treatment with the patient, i.e. is the patient adequately treated for his/her current conditions. The GP and patient assess whether treatment adjustments are needed. The GP explores if anything discussed during the consultation requires follow-up, i.e. referrals to the municipality, a psychologist, “institutional care facility” or other services listed by the SOFIA handbook. The GP creates a safety-net – by emphasizing that the patient is always welcome to contact the practice. If medically indicated, paraclinical tests and follow-up consultations will be scheduled.

The extended consultation should be conducted by a GP and was fully reimbursed financially. The extended consultation aimed to ensure patient-centeredness and shared-decision making in planning care, e.g. ending the extended consultation with a shared care plan. It also included a focused health examination and medication review by the GP.

In developing this intervention, we adopted a patient-centred care approach. Patient-centred care can be defined as care that elicits individuals’ values and preferences. Once expressed, it allows this to guide all aspects of the individual’s healthcare, supporting their realistic health and life goals [17, 18]. German philosopher Martin Buber propagated in the 1920s the need for doctors to approach patients in a patient-centred manner. Buber called for a shift in focus from the disease to, first and foremost, attending to patients as human beings [19]. In general practice, we find patient-centred care to be the practice of caring for patients in ways that are meaningful and valuable to the individual patient [20]. Patient-centred care relies on the GPs’ clinical expertise in examination, diagnostics, and treatment decisions and on the GPs’ ability to involve the patients’ narrative, i.e. personal preferences and values in the consultation [21, 22]. In Denmark, general practice is known for continuity- and patient-centred care [23]. However, many find patient-centred consultations challenging to conduct with patients with SMI (Jønsson ABR, Svanholm SL, Brodersen JB, Reventlow S, Brostrøm M: General practitioners’ experiences of providing somatic care for patients with severe mental illness: a qualitative study, forthcoming).

### Need for feasibility testing of the preliminary intervention

When presented with the SOFIA intervention design, several GPs, researchers, and stakeholders voiced concerns that general practice would not have the time and personnel to introduce extended consultations into routine care. It was also unclear how patients would experience being invited to an extended consultation on the GP’s initiative and how they would experience the consultation. Furthermore, many trial-related aspects concerning the feasibility of conducting a trial in general practice were unknown, including how to identify, eligibility screen, and recruit patients.

Therefore, we wanted to study these key uncertainties related to the feasibility of introducing an extended consultation in general practice to patient with SMI. Thus, the aims of the feasibility study were threefold: 1) to investigate how GPs experienced the feasibility of introducing an extended consultation for patients with SMI, 2) to assess the clinical content of extended consultations and how these were experienced by patients with SMI, and 3) to investigate the feasibility of identification, eligibility screening, and recruitment of patients with SMI.

## Methods

### Design

This study was a single-arm intervention study. In keeping with other studies, we use the term feasibility study as an umbrella term for any study that aims to support the development of a future study [24–26]. Since we primarily used qualitative methods to assess the study aims, we report its methods and findings using relevant items from the CONSORT extension for Pilot and Feasibility Trials [27] and the COREQ criteria for qualitative research [28] (Additional file 1).

### The study in the context of other complex intervention phases of the SOFIA project

The study was planned to last 6 months from mid-January 2020. However, the study was terminated ahead of schedule by the end of March due to the Covid-19 pandemic. We could conclude from the preliminary data collected during the study that a more rigorous and comprehensive pilot study was needed. We found that many aspects concerning the feasibility of the intervention, and its implementation in general practice, were suboptimal or needed more extensive investigation, e.g. how to ensure fidelity to the preferred delivery of the intervention. Using the evidence obtained in this feasibility study, we planned the design of a subsequent pilot study [14].

### The context of general practice in Denmark

In Denmark, GPs are organized within local groups (Danish: klynger) that collaborate on supervision, courses, and small-scale local quality improvement projects relevant to their everyday clinical practice [29, 30]. One group of GPs from the northern part of Jutland approached the SOFIA project group for advice about a local quality improvement project they were planning to improve care for patients with complex multimorbidity [31]. The GPs and the SOFIA project group agreed to collaborate on patients with SMI as an exemplary case for caring for patients with complex multimorbidity in general practice. The GPs received support for improving their quality of care for patients with SMI by participating in the SOFIA feasibility study, i.e. agreeing to participate in interviews, following formal eligibility screening criteria and other trial-related tasks.

### Setting and participants

**Fig. 1:**
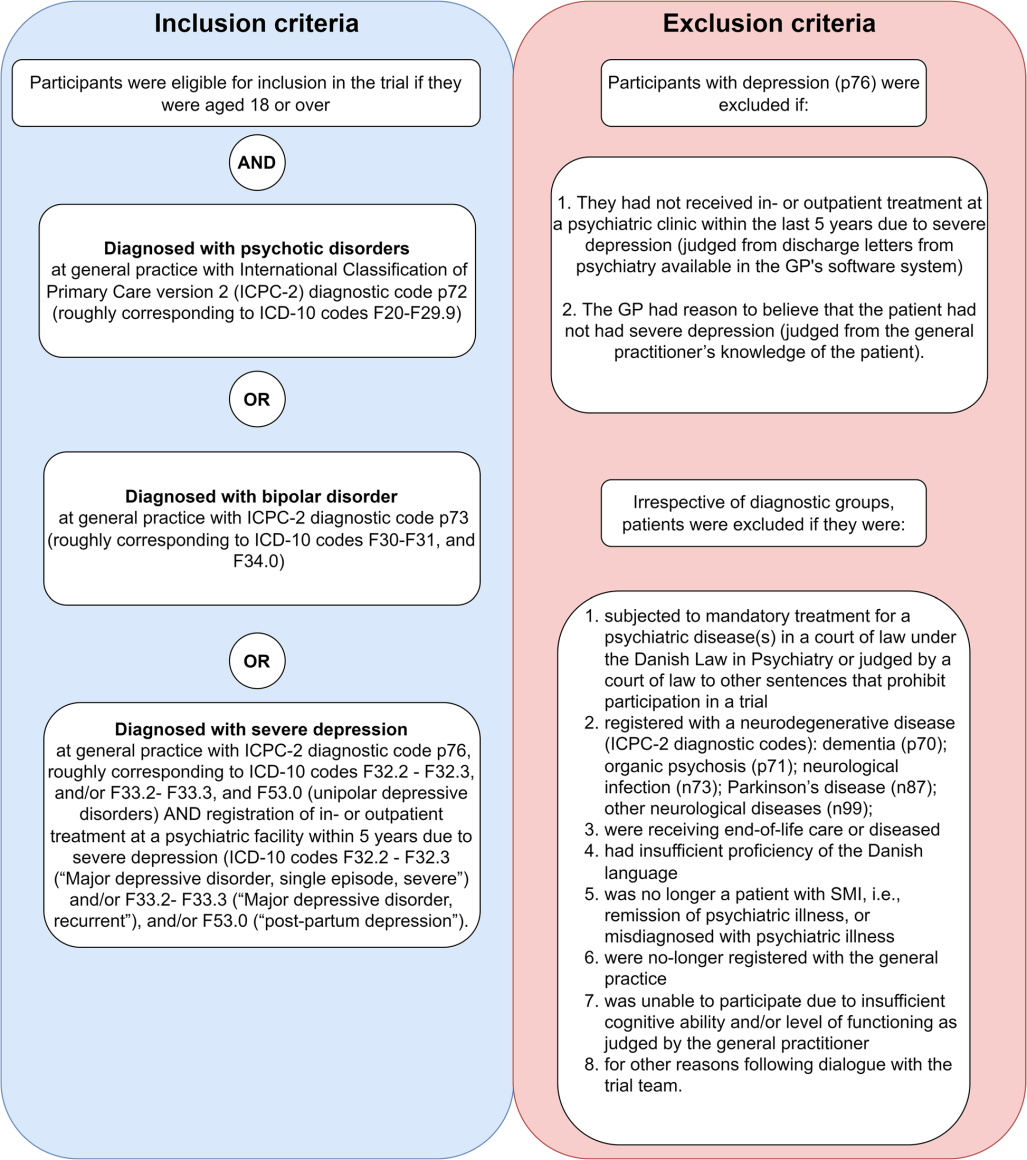
Patient eligibility and selection of patients according to their main SMI diagnosis

Patients with SMI were identified from patient records using the diagnostic system International Classification of Primary Care version 2 corresponding to psychotic disorders, bipolar disorder or severe depression (Fig. 1). In each general practice, the GP screened the patient record for eligible patients using the eligibility criteria (Fig. 1). Subsequently, practice personnel contacted eligible patients by phone and invited them to a 10-min conversation in the general practice. Here, practice personnel informed them about the study, and informed consent was obtained if the patient was eligible.

### The intervention

**Fig. 2:**
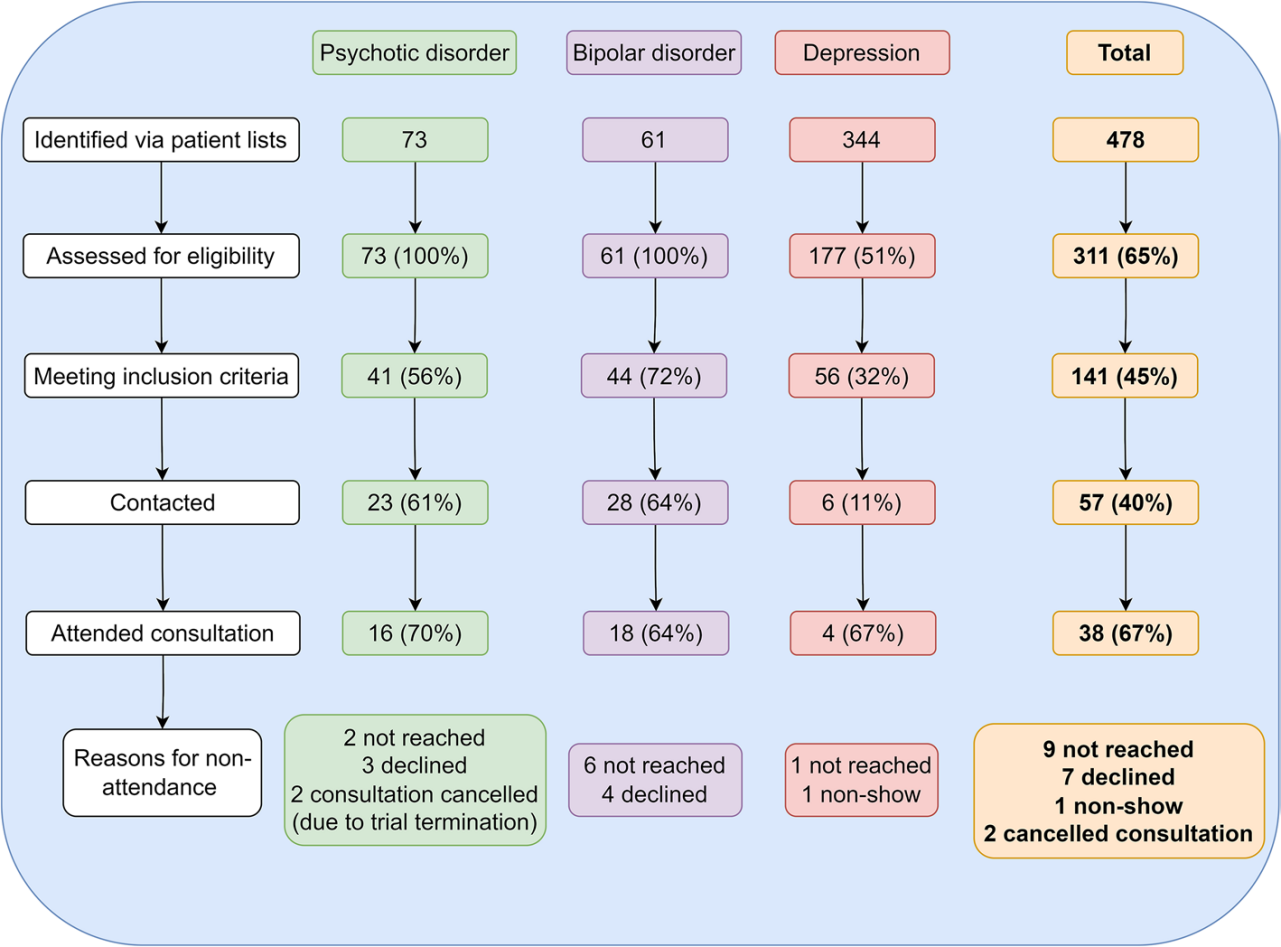
Patient flow chart in general practices

GPs attended a 3-h introductory course, defining the start of the study. Here, the intended conduct of the extended consultation was discussed with GPs to promote fidelity. The GPs also received guidance on carrying out study-related tasks, i.e. identifying, eligibility screening, and recruiting patients, and documenting these tasks and the clinical outcomes of the extended consultation. GPs received written materials to ensure correct documentation and guidance during the consultation. Following the introductory meeting, the staff at each general practice made a list of potentially eligible patients (see inclusion criteria in Fig. 1), which GPs subsequently screened for eligibility (see Fig. 2). Patients agreeing to participate in the study were asked to fill out one survey concerning the generic quality of life (the EQ5D5L survey [32]) and one survey on needs and preferences relating to life quality [33], which, if permitted by the patient, could be discussed during the consultation with the GP.

#### The extended consultation

The consultation was to comprise up to 45 min and follow the SOFIA scheme (Table 1) inspired by the patient-centred consultation model [34], divided into a patient part, a practitioner part, and a shared part. This consultation model intended to permit time for the patient to express their illness experiences, preferences and values, a dialogue about the patient’s quality of life, and a focused physical examination personalized to the patient’s medical history and complaints alongside a medication review. The consultation concluded with a dialogue between the GP and the patient about further potential diagnostic follow-up and, if relevant, changes to current treatment or referral to specialists or services in the municipality, i.e. shared decision-making. GPs were to schedule follow-up appointments as needed.

### Data collection

The study evaluated the feasibility and content of the extended consultation from qualitative and quantitative data. In each general practice, three types of case report forms were filled out. Practice personnel filled out the first and second case report forms, which contained information about the number of patients recruited for the study and the characteristics of study participants, respectively. The GPs filled out the third case report form, which contained information about changes to the care plan following the extended consultation (Table 2).

**Table 2:** Case report forms

Case report form 1 (Recruitment of study participants), filled out by practice personnel:
1) Number of patients assessed for eligibility
2) Reasons for excluding patients
3) Number of patients meeting inclusion criteria
4) Number of patients contacted and offered an extended consultation
5) Number of patients attending an extended consultation
6) Reasons for non-attendance by patients
Case Report Form 2 (Characteristics of study participants), filled out by practice personnel
1) Sex
2) Age
3) Other diagnoses than SMI
4) Living with partner (yes/no)
5) Job status
6) Number of appointments last year with the GP
Case report form 3 (Changes to the care plan following extended consultations), filled out by the GP
1) Medication changes
2) Changes in diagnoses
3) Referrals
4) Other care in practice
5) More frequent care in practice

A medical anthropologist (MS) observed the recruitment process in general practices and conducted interviews and informal conversations with GPs and staff. Extended consultations were observed and field notes made by MS following the provision of consent by both GP and patient, and whenever possible, given that several extended consultations took place simultaneously across general practices. In total, 20 (53%) extended consultations were observed. Additionally, 98 h of observation were conducted in general practice during the study period. This part of data collection focused on the clinic’s organization and everyday work life and how extended consultations were made to fit into this setting.

All participating GPs were interviewed following a semi-structured interview guide, and patients were additionally informally interviewed concerning their experiences with the extended consultation. Repeat interviews were carried out as needed. All interviews lasted between 9 and 25 min. Additionally, a 2-h focus group was conducted, entailing the use of the fishbowl technique [35] and plenum discussion. Here, all participating GPs and junior general medicine doctors participated, and AJ, JBB, MS and FM facilitated the focus group.

Interviews were audio-recorded, and observations were documented via field notes. Both were structured via an interview and observation guide, respectively. These covered four main themes concerning the intervention: (1) Experiences and perceptions of the extended consultation; (2) Challenges arising when conducting the extended consultation; (3) Content of the consultation; (4) Relevant changes in treatment or care planning following the extended consultation and four themes concerning feasibility of conducting the trial: (1) General information about the practice, (2) Recruitment and the patients included in the project, (3) Patient access to the practice, and (4) Introduction to the study and data collection. The guides (Additional file 2) were adapted and adjusted pragmatically to fit the particular context and GPs or patients involved, i.e. regarding practicalities or themes arising during the study.

### Analysis

MS and a student worker transcribed all interviews. Transcripts were not returned to GPs or patients for comment or correction. MS and AJ analyzed transcripts and field notes following an interpretative phenomenological analysis (IPA) [36] allowing for a detailed examination of the main questions in the study. The IPA method is particularly valuable in this setting as it enables the micro-level reading of participants’ experiences [36]. IPA allows for detailed analysis of formal interviews, informal talks, and field notes before moving to more general claims, ensuring that all participants are equally heard. Transcripts were coded by MS and subsequently grouped under three main themes: Experiences with extended consultations, challenges related to conducting the extended consultations, and content of the extended consultation. We intended to provide participants the opportunity to give feedback on the findings. However, due to early termination because of the COVID-19 pandemic, this was not possible. AJ translated the informants’ quotes into English. NVivo12 software was used to manage the data.

## Results

Before the study commenced, seven general practices expressed interest in participating. Two general practices withdrew participation, one due to time constraints and one due to illness among practice staff. The five remaining general practices employed seven GPs (Table 3).

**Table 3:** Characteristics of the five general practices participating in the study

**Practice**	**Number of general practitioners, N**	**Total number of patients registered at the practice, N**	**Number of eligible patients with SMI, N (%)**
**1**	2	3200	39 (1.2)
**2**	1	1600	62 (3.9)
**3**	1	1600	78 (4.9)
**4**	1	1800	109 (6.1)
**5**	2	3116	190 (6.1)

### Feasibility of introducing extended consultations for people with SMI in general practice

We identified 478 patients with SMI. Of these, 73 (15%) were diagnosed with a psychotic disorder, 61 (13%) had bipolar disorder, and 344 (72%) had depression. Across the three diagnostic groups, 141 (45%) patients met the inclusion criteria. Due to the early termination of the study caused by the COVID-19 pandemic, only 57 (40%) of the 141 eligible patients were contacted. Of these, 41 (72%) agreed to participate, but one patient did not show up, and two cancelled their appointment. Thus, of those contacted, 38 (67%) attended an extended consultation (Table 4).

**Table 4:** Characteristics of patients participating in the trial

**Diagnosis**	**Other diagnoses**	**Any change to care**	**Medication change**	**Diagnostic changes**	**Referral**	**Other**
**Bipolar disorder**	Asthma	No				
**Bipolar disorder**	COPD	Yes				Monthly follow-up with GP
**Bipolar disorder**	Epilepsy	Yes				Wants to discontinue follow-up in neurology
**Bipolar disorder**	Hypertension, hypercholesterolemia, chronic kidney disease	Yes				
**Bipolar disorder**	Idiopathic urticaria	Yes				More frequent follow-up
**Bipolar disorder**	Myxoedema, asthma, chronic back pain	No				
**Bipolar disorder**	Myxoedema, fibromyalgia, depression, ADHD	Yes			Back pain	
**Bipolar disorder**	None	No				
**Bipolar disorder**	None	Yes		Anaemia		Follow-up testing
**Bipolar disorder**	None	Yes				Blood samples testing
**Bipolar disorder**	None	Yes				More frequent follow-up
**Bipolar disorder**	None	Yes				More frequent follow-up
**Bipolar disorder**	None	Yes				More frequent follow-up
**Bipolar disorder**	Parkinson’s disease	Yes			Psychiatry and Municipality	
**Bipolar disorder**	Rheumatoid arthritis, laryngeal cancer	Yes				More frequent follow-up
**Bipolar disorder**	Unknown	No				
**Bipolar disorder**	Unknown	Yes			Municipal service offer	
**Bipolar disorder**	Unknown	Yes				Considering diagnostic changes
**Depression**	Asthma, Hypertension	No				
**Depression**	Alcohol dependency/ misuse	Yes				More frequent follow-up
**Depression**	Polyneuropathy	Yes	YES			
**Depression**	None	Yes			Orthopaedic surgery	
**Psychotic disorder**	acne, unspecified personality disorder	No				
**Psychotic disorder**	Anxiety and depression	Yes			Psychiatry	
**Psychotic disorder**	Anxiety	Yes			Psychiatry	
**Psychotic disorder**	Asthma, hypercholesterolemia, back pain	Yes			Lung medicine	Contact the municipality for more intensive care
**Psychotic disorder**	Congenital cerebral palsy, depression	No				
**Psychotic disorder**	COPD, diabetes, psoriasis	Yes			dentist	Follow-up lifestyle
**Psychotic disorder**	Type 2 Diabetes mellitus	Yes				Frequent follow-up for psychological problems
**Psychotic disorder**	Hypertension	Yes	YES			Follow-up each 6 months
**Psychotic disorder**	None	Yes				Weight loss program in general practice
**Psychotic disorder**	Sarcoidosis	Yes	YES			
**Psychotic disorder**	Unknown	Yes			Liver specialist	Reduce alcohol use, test liver function
**Psychotic disorder**	Unknown	Yes	YES			
**Psychotic disorder**	Unknown	Yes				Reduce cannabis use
**Psychotic disorder**	Unknown	Yes				Unclear
**Psychotic disorder**	Unknown	Yes				Home visit
**Psychotic disorder**	Unknown	Yes			Municipal service offer	

Of the 38 patients receiving an extended consultation; 17 were females (45%), 21 were males (55%). The median age was 48.5 years with an interquartile range of 19.75 years

Nine patients could not be reached, and seven declined participation (Fig. 2).

### Experiences with extended consultations

In informal conversations before the study commenced, GPs expressed feeling overwhelmed by the amount of information provided during the introductory meeting. In addition, GPs expressed concerns that 45 min allocated to a single consultation would prove too long for patients. Moreover, since consultations in general practice usually last 10 to 15 min, the GPs anticipated that introducing the extended consultation into practice would give rise to logistical problems. In practice, all GPs found it possible to introduce extended consultations into routine care, although opinions diverged concerning possible disadvantages to other patients. Here, some GPs voiced concerns that spending additional time on a few selected patients came at the cost of other patients at the clinic due to the limited time available to GPs.

The GPs’ initial concern about whether patients with SMI would be capable of enduring an extended consultation proved unfounded. Instead, extended consultations with GPs appeared to elicit patients’ interest:

*Well, I found it quite easy just to call them [patients] (…) the majority just said yes (…) so it’s been going really well (…) and it is the possibility of seeing the doctor for a longer consultation that has been attracting the patients (Resident in general medicine, focus group)*

From conversations with patients, it was clear that patients found that the extended consultation was beneficial, noting that it indicated that the healthcare system was prioritizing their concerns, which the participating patients recounted that they had rarely experienced. One patient used the extended consultation as an opportunity to hand the GP a plastic bag containing books and notes that he felt the GP ought to see to understand him, which was important to him (Field note, March 2020). In informal conversations, patients expressed that it made a positive difference in their healthcare that they were a) invited to come and thus did not feel that they were taking up unnecessary time and b) that they could address more problems in the extended consultation as opposed to regular consultations. Patients also expressed an interest in participating in research, given the possibility of improving their health and helping other patients with SMI. Two patients (5%) described a negative experience with the extended consultation, experiencing that their GP was unwilling to address their concerns, strengthening their belief in the futility of consulting their GP when they had health problems.

### Challenges related to conducting extended consultations

Conducting extended consultations presented challenges. Despite instructions shared at the introductory meeting and in the study pamphlet, in observed consultations, GPs faced difficulties adhering to the SOFIA scheme:

*“Because we just do as we’re used to, right? Well, you get this antipsychotic and then we do this and this. And then I would be like, and how are things? I mean, you always pose these questions (…) but this is different” (GP7)*

Also, the instructions in the SOFIA scheme were met with some reluctance:

*“I don’t think it makes any sense that I have to use the stethoscope (…) It [the stethoscope] has little to no sensitivity and specificity, it’s more of a symbolic act that we do. So well, I’d rather write [in the SOFIA scheme] that it’s an option, not a requirement” (GP1)*

Practical challenges also arose in the conduct of extended consultations. Critical tasks in general practices meant that extended consultations were often interrupted with questions or comments by staff or medical residents. This was perceived as frustrating by both GPs and patients.

Cancellations and patient no-shows likewise proved challenging. Two consultations were cancelled, and one patient did not attend a planned consultation (See Fig. 2). One patient cancelled the appointment for the extended consultation at the last minute leading to frustration from the GP about the concept of an extended consultation due to the “loss of” 45 min in the context of a tight schedule. Two GPs managed the risk of non-attendance by planning extended consultations either at the end of the day, when a no-show would be less inconvenient or during morning hours, when non-attendance would leave room for acute patients. Management of non-attendance resulted in overall greater satisfaction with the intervention among these GPs. However, placing an extended consultation, during which GPs and patients often discussed quite complex issues, at the end of the day also proved difficult for a GP on one occasion, leading to the GP ending the consultation before time as she had difficulty discussing complex issues that late in the day.

### Content of the extended consultation

All GPs agreed that the duration of the extended consultation both enabled patients to set the agenda and led to a discussion of complex challenges faced by patients formerly unknown to the GP:

*“It has been four very different consultations, with different themes. It could be loneliness, it could be work-related issues, and then one of them [patients] just needed to talk. And talk and talk and talk. And from that arose physical challenges or complaints. So there’s been more than enough to discuss, and I’ve also had the feeling that they [patients] have been very happy that they felt time had been allocated to them contrary to a usual consultation which lasts maybe 10 min, right?” (GP6, focus group)*

Another GP expressed how the allocation of additional time allowed for further elaboration of the individual aspects of patients’ challenges:

*“In my experience, so many of these conversations with patients are about being lonely or not being able to be there for one’s loved ones. That’s usually what bothers them the most (…) and then we try to locate the roots of that feeling. Sometimes it’s just regular physical challenges that we just never have discussed. For instance, one is incontinent and wearing a diaper. So she is reluctant to see other people, and [GP realizes] ‘we never made that gynaecological exam’ (…) or another one having severe trembling making her fall on the street, and she feels so sad because it prevents her from going to dances, which is her only hobby. She’s 70 (…) so I decide to get that checked, is it side effects of her meds or is it her Parkinson’s disease which is developing fast?” (GP5)*

Hence, the structure of the SOFIA scheme allowed for discussion of whatever was experienced by the patient as a symptom, sensation, or challenge. Elicitation of patient preferences and values took up most of the agenda for the extended consultation. For example, one consultation included a discussion of family dynamics, identity, and blood pressure (P11), another focused on alcohol treatment (P1), and yet another consultation centred around ways of engaging in exercise while also revealing that the patient wished for increased continuity of interaction with the GP (P13).

Notwithstanding what the GPs perceived to be benefits of the extended consultation, they found it challenging to adhere to the SOFIA scheme, especially regarding allowing the patient to set the agenda for the conversation. In as many as eight consultations (21%), it was observed that the GP struggled with the task of allowing patients’ values and needs to guide the consultation. Instead, many GPs set the focus of the conversation in line with their perceptions of what mattered most. For instance, one GP dismissed a patient’s complaint concerning conflict with social workers as out of their hands (P20). In another example, a patient’s primary concern related to a physical ailment that the GP deemed benign and temporary, steering the conversation towards other issues, causing great frustration for the patient (Field note, February 2020).

During observed consultations, most GPs did not perform focused physical examinations of the patients as intended. Instead, prefilled surveys about patients’ quality of life unintendedly became the centrepiece of the consultation in place of somatic symptoms and concerns. Based on observations, none of the GPs used the recommended algorithm for medication review nor materials to support adherence to the SOFIA scheme, i.e. guiding material on how to structure and conduct the extended consultation.

### Changes to the care plan after extended consultations

Overall, 31 of the 38 patients attending the extended consultation (82%) experienced at least one change in their treatment or care as a direct result of the extended consultation (Table 5).

**Table 5:** Health care provision for patients with SMI following the extended consultations

	**All patients (*N* = 38)**	**Psychotic disorder (*n* = 16)**	**Bipolar disorder (*n* = 18)**	**Depression (*n* = 4)**
**Any change to care, n %**	31 (82)	14 (88)	14 (78)	3 (75)
**- medication changes, n (%)**	4 (11)	3 (19)	0 (0)	1 (25)
**- changes in diagnoses, n (%)**	1 (3)	0 (0)	1 (6)	0 (0)
**- referrals**^**a**^**, n (%)**	10 (26)	6 (38)	3 (17)	1 (25)
**- other care in practice, n(%)**	20 (53)	9 (56)	10 (56)	1 (25)
**- more frequent care in practice, n (%)**	12 (32)	4 (25)	7 (39)	1 (25)

- Categories are not mutually exclusive; e.g. one patient could both be referred and change medication

^a^When one patient had more than one referral, only one of the referrals count

Changes to healthcare provision included scheduling blood samples or other diagnostic procedures for somatic health concerns, follow-up visits concerning mental health problems, recommendations concerning lifestyle changes, and help reducing substance use. 26% of patients were referred to other specialist health care providers or social care. Reasons for referral included assessing lung function at a pulmonologist, medication or diagnostic assessment at a psychiatrist, and requesting more support for the patient from the municipality.

## Discussion

### Summary of main findings

We found that extended consultations were feasible to plan and execute with acceptable procedures for identification, eligibility screening and recruitment of patients and relatively high participation from patients, except for a few occasions when patients did not attend the consultation. This finding contradicted concerns initially expressed by GPs relating to the practicalities of time constraints in-clinic and patients’ capacity to endure such long consultations. All GPs agreed that extended consultations on several occasions demonstrated the possibility of enabling increased consideration of patients’ individual preferences and values. In 82% of consultations, patients had a change in their health care plan. These changes were primarily scheduling follow-up visits and referrals to other specialists or care in the municipality. The effects of these changes on the care of the health of study participants require studies with a longer follow-up time. Regrettably, we could not assess whether the feasibility or conduct of extended consultations would change over time, e.g. potential improvements in fidelity due to training, due to the early termination of the study because of the COVID-19 pandemic.

Introducing extended consultations in general practice was not without logistical challenges. For example, three patients (8%) either cancelled or did not attend the extended consultation. General practitioners and patients were frustrated when consultations were interrupted due to other patients with acute health problems, which required the GP’s immediate attention. Another concern for GPs was whether extended consultations might come at the cost of other patients, something we could not assess in this study. Regarding adherence to the SOFIA scheme, it appeared from observations of extended consultations that most often, GPs did not perform a physical examination as intended, nor did they use the recommended algorithm for medication review and materials to support adherence to the SOFIA scheme. Following this, and that GPs had felt overwhelmed by information on the introductory seminar, we see a need for a full-day course before GPs execute this intervention. Despite the low fidelity to the SOFIA scheme, both GPs and patients found that the consultations were beneficial. Despite the co-design phase, our data show the need for feasibility studies. This owes to the limitations of co-design as being discussed with selected participants in workshops, whereas this feasibility study have tested the intervention in real-life settings in general practice.

### Strengths and limitations

The study has several strengths. First, the study’s rationale and design are well-grounded in theory and based on a 2-year co-design process involving patients and key stakeholders [37, 38]. Second, as the intervention was tested in everyday general practice settings, findings likely indicate those to be encountered should the intervention be introduced into routine general practice. Third, the study was conducted in line with the recommendation of the Medical Research Council [26], advocating for a stepped approach to trials of complex interventions. Fourth, we used multiple data sources, i.e. field notes, interviews with both GPs and patients and a focus group with GPs, which strengthens our information power for the interpretations drawn in this publication [39].

The study’s main limitation was its premature termination due to the COVID19 pandemic, limiting the number of patients included and shortening the follow-up time after patients’ extended consultation. This premature termination of the study hindered the assessment of potential changes regarding the feasibility of introducing extended consultations in general practice and potential improvement in fidelity to conduct of the consultations, i.e. adherence to the SOFIA scheme. Secondly, the premature termination of the study limited the time that general practices had to adopt the intervention and adapt it to their local usual-care practices. More time would possibly have led to the improved conduct of extended consultations, i.e., fidelity to the SOFIA scheme. More time would also have allowed for investigating whether the GPs’ and the patients’ views about extended consultations would change over time.

Furthermore, general practices had to cancel in-person follow-up consultations after the initial extended consultation due to the pandemic and resulting lockdown. This arguably had a negative influence on the introduction of extended consultations into routine care, both in terms of adaptation to the new work routines around the consultations and concerning the patients’ experiences of the value of the consultations. Thirdly, our study was based on supporting GPs in their local quality improvement project, simultaneously getting insight into how extended consultations would be implemented into routine care by simply introducing the concept and providing information about the recommended conduct of such consultations. As the GPs and practices who participated in the feasibility study also needed instruction on how to report data to the research team, a substantial amount of the instructional meeting was spent on how to accurately report data. We believe this contributed to the low fidelity to the intervention, as more detailed information on the SOFIA model was then only distributed in a written format, which most of the GPs likely did not find time to read. We would argue that a more detailed and rigorous implementation strategy, e.g., hosting training courses, providing videos or other examples of preferred conduct, having supervision practices set up and the like, would have increased fidelity to the extended consultation since GPs did find that intervention acceptable and meaningful for patients. In effect, changes to healthcare demonstrated in our study are likely conservative in terms of the true potential effects of these consultations. Fourthly and to the contrary, the GPs recruited for the trial represents a selected sample of GPs that were interested in the topic and on their own initiative had made contact to the SOFIA research group, which arguably reduces the generalizability of findings, and perhaps could lead to issues with reproducing the acceptability of the intervention in future studies.

### Possibilities for patient-centred care in extended consultations

Results show that extended consultations provide a suitable setting for attending to patients’ individual preferences and values, both in terms of the content and conduct of the extended consultation and regarding the shared care plan following the discussion. It also allows GPs to accommodate multiple problems in parallel, and GPs may receive adequate guidance on conducting extended consultations, e.g. proper dialogue with GPs about how to ensure fidelity to the SOFIA scheme [14]. Here, we understand values as referring to a moral and cultural orientation and preferences as personal feelings towards different treatment options for mental and somatic illnesses [40]. As shown, the extended consultation allowed patients’ various concerns to be addressed, even when the concern was more social than medical, as a starting point for the consultation. In contrast with regular consultations, the extended time frame allowed attention to somatic, mental, and social concerns.

One of the most articulated dimensions of patient-centred care is the therapeutic relationship [20], which builds on a constructive relationship, prioritizing respectful communication between patient and physician [41]. We saw such therapeutic relationships enacted in several extended consultations when GPs followed instructions, allowing patients’ perspectives to guide the plan.

Our data underline the importance of eliciting patients’ individual preferences and values, given that life circumstances influence patients’ experiences of their illness and treatment [42, 43]. Our data showed large variation in what mattered to patients and thus the importance of allowing patients to set the agenda, which allowed patients to bring their life stories and circumstances into the conversation to determine what matters most to the individual patient and investigate sensations and symptoms that might indicate underdiagnosed or undertreated somatic illnesses. Most patients with SMI are managed poorly with usual primary care arrangements due to their significant and complex social and health challenges [44, 45] and a general unequal provision of care, emphasizing the importance of patient-centred care [46, 47]. This calls for a patient-centred approach encompassing life circumstances and patient perspectives reflected in a constructive therapeutic relationship. Another important dimension of patient-centred care is the biopsychosocial holistic approach [20], in which care is thought to encompass all domains of health, i.e. biophysical, cognitive, emotional, behavioural, and social domains [41].

Last, it should be noted, that while the extended consultation in the SOFIA scheme must be done by a GP owing to the possible need for diagnosing new conditions, follow-up consultations and yearly controls could be executed by other healthcare professionals, e.g. some practices have well-functioning relations between patients with SMI and the practice nurse, and the nurse already perform some of these consultations for already known co-morbidities in the patient.

### Implications for practice

This feasibility study demonstrates that conducting extended health consultations for patients with SMI in the general practice setting is feasible. However, we also offer several suggestions for improved future implementation. A core premise for conducting extended consultations in general practice is allocating sufficient time. One worry is the current scarcity of GPs in Denmark, already burdened by a large patient load [48, 49]. Based on the above mentioned results and discussions, we hypothesise that extended consultations with patients in marginalized or vulnerable positions may in the long run release time as complications from undertreated and underdiagnosed conditions can be avoided. Extended consultations may also mitigate the inverse care law, by giving more time to those most in need [50]. This hypothesis require further investigation. In addition, time is also necessary for patient-centred care and a well-functioning patient-GP relationship [51, 52]. Yet, time constraints are typical for general practice care. Introducing extended consultations into general practice, therefore, does also require a cultural change in regards to the time allocated to individual patients. Importantly, such changes also require sufficient reimbursement for the resources required in the collective efforts related to introducing extended consultations into general practice, e.g. identifying patients, screening potentially eligible patients, contacting eligible patients, and conducting extended consultations and resulting follow-up measures.

Our findings also highlight the importance of ensuring that various implementation strategies are used to ensure fidelity to the intervention, e.g., providing clear instructions and engaging GPs in a dialogue about the intended conduct of the extended consultation, i.e., using the SOFIA scheme. Thus, for future extended consultations and future studies, it is recommended to host a comprehensive introductory course to ensure fidelity to the intended conduct of the extended consultation, i.e., fidelity to the SOFIA scheme (Table 1). This point relates to the generally well-accepted value of involving a diverse range of stakeholders when co-designing complex interventions [53].

It has been suggested that physical exams should be performed as a routine component of extended consultations for patients with SMI [54]. This group is less likely to be offered medical investigations, tests, or referrals for further check-ups [55–57]. We found that many GPs did not perform physical exams during extended consultations, although this was the intent of the SOFIA scheme (Table 1). This suggests that implementation strategies are needed to promote this change in usual care. However, it will be important to find the right balance between allowing GPs to make and act upon clinical judgment while ensuring fidelity to the essential elements of the extended consultation, e.g., physical examination, commonly referred to as promoting fidelity in function while allowing variance in the form of the intervention [58].

It is paramount for the feasibility of introducing extended consultations in general practice that patients are willing to participate, and that practice personnel can contact and recruit them for the intervention. Our findings regarding acceptable procedures for identification, eligibility screening and recruitment of patients and the relatively high participation rate of patients that can be hard to reach, and few cancellations and “no-shows”, suggests an acceptable clinical feasibility of implementing our patient recruitment strategy in general practice.

### Future research

Due to the small size of this study and its premature termination, we cannot conclude whether we have succeeded in recruiting “the right patients”, i.e. those patients who will benefit from the intervention. This question will be further investigated in future trials [14]. Future research should investigate the direct and indirect costs of introducing extended consultations for selected “high-risk patient groups”, e.g. people that are marginalized, vulnerable, and often have complex care needs, as the provision of more time for some patients might come at the expense of other patients. Additionally, we found that most, but not all, patients appreciated being contacted and taking part in the study. Hence, future research should investigate the potentially negative effects of contacting vulnerable patients and how these can be mitigated. Building on the findings from this feasibility study, other aspects related to the feasibility of the SOFIA intervention will be pilot tested in a cluster-randomized two-arm pilot trial [14].

## Conclusion

The findings of this study show that it is feasible to introduce extended consultations for patients with SMI in general practice, as evidenced by high participation rates, with the majority of contacted patients agreeing to participate in the study and only three patients not attending the extended consultation. General practices and patients with SMI additionally found this type of intervention acceptable. Although extended consultations were described as well-suited to eliciting patients’ values and preferences, challenges were identified related to the GPs’ adherence to the SOFIA scheme for the conduct of the extended consultations, marking the importance of proper guidance for GPs. Finally, we conclude that general practices require financial reimbursement for the time allocated, and also in the case of patient non-attendance, to make extended consultations financially feasible for general practitioners.

### Supplementary Information

**Additional file 1.**

**Additional file 2.** Interview and observation guide.

## Data Availability

The dataset(s) supporting the conclusions of this article are included within the article, following guidance on preparing raw clinical data for publication [59].

## Abbreviations

CONSORT: Consolidated Standards of Reporting Trials
COREQ: Consolidated criteria for reporting qualitative research
EQ5D5L: EuroQol 5 Dimension 5 Level survey
GP: General practitioner
IPA: Interpretative phenomenological analysis
KEU: An acronym in Danish for “Kvalitets- og EfterUddannelsesudvalget”, directly translated it means the Committee for Quality and Continued Education
N: Number
P: Patient
SMI: Severe mental illness
SOFIA: An acronym in Danish for”*S*ammen *O*m *F*ysisk og psykisk helbred *I**A*lmen praksis, directly translated it means (working) Together for Physical and Mental Health in General Practice
