# Supplementary material for: Introducing extended consultations for patients with severe mental illness in general practice: Results from the SOFIA feasibility study
Source: BMC Prim Care. 2023 Oct 5;24:206. doi: 10.1186/s12875-023-02152-z (PMC10552249; doi:10.1186/s12875-023-02152-z)
Supplement: Supplementary file 1 — Additional file 1. [file 12875_2023_2152_MOESM1_ESM.docx]

# additional information

## Characteristics of interviewers and facilitators

During the feasibility study, MK (she), a medical anthropologist, was employed as a research assistant at the SOFIA project. AJ (she), PhD, medical anthropologist, was employed as a postdoc at the SOFIA project, having 12 years of experience conducting solo and focus group interviews. JBB (he), PhD, general practitioner, was employed as a professor in general practice and affiliated with the SOFIA project. JBB has been teaching and facilitating small-group evaluations for the past 20 years. FM (he), a medical doctor, was employed as a trial manager for the SOFIA trial.

## Relationship with participants

The general practitioners (GPs) within the research group from Denmark, i.e., SR, AM, and JBB, knew one of the GPs before the commencement of the feasibility study, having collaborated on other projects beforehand. The six other GPs participating in the study did not have any relationship with the research group before study commencement. None of the participating patients (N=38) knew the research group beforehand.

Interviewer characteristics: GPs and patients were told that the researchers collecting data, i.e. MS and AJ, via fieldwork, interviews and focus groups were interested in understanding how they experienced the intervention to refine it for future studies further.
