# Supplementary material for: Introducing extended consultations for patients with severe mental illness in general practice: Results from the SOFIA feasibility study
Source: BMC Prim Care. 2023 Oct 5;24:206. doi: 10.1186/s12875-023-02152-z (PMC10552249; doi:10.1186/s12875-023-02152-z)
Supplement: Supplementary file 2 — Additional file 2. Interview and observation guide. [file 12875_2023_2152_MOESM2_ESM.docx]

Interview and observation guide

# Guide for Interviews and Observations of Extended Consultations

## 1) Experiences and perceptions of the extended consultation

- How much time is set aside for the patient?
- What is the attitude towards this time - doctor/patient? Is it always with the doctor, or are there practices where the nurse is the primary person? Possible reasons for the attitude (e.g., lack of time, patient preference). What is the patient’s preference in regards to who should be the primary person?
- How is the time spent in the consultation perceived by both the patient and the doctor? Is it considered appropriate?

## 2) Challenges arising when conducting the extended consultation

- What is the consultation process like? Is it typically followed as planned, or are there deviations? If so, what are the reasons for the deviations?
- How is the health check conducted? Are there physical examinations, lab tests, or other procedures involved? When are they performed? Does the doctor respond physically to the patient's symptom descriptions if any?
- How is the consultation itself prioritized? Are the patient's self-described needs included, and does the patient have a voice?
- What is the focus of the consultation? Is there an emphasis on a risk approach or a resource approach?
- How are patients when they arrive for the extended consultation?

## 3) Content of the consultation

- How is the patient's body language during the consultation? What non-verbal cues can be observed?
- How is the doctor's body language in these consultations compared to ‘regular’ consultations? Is there consistent eye contact? How is the doctor using the computer during the consultation? How do they use the questionnaire about life quality competed before the consultation during the consultation?
- At the end of the consultation, is there a plan? Do the patient and doctor agree on it?
- What follow-up actions are agreed upon?
- How do the patients react during the extended consultation in terms of statements, actions, emotions?

## 4) Relevant changes in treatment or care planning following the extended consultation

- How is the overall experience of the extended consultation perceived by both the doctor and the patient?
- Do they utilize contact with psychiatric services during the consultation?
- Is the medication review template used, and if so, how?
- Are referrals made to municipal organizations or other relevant services?

# Guide for Interviews and Observations of Trial feasibility

## 1) General info about the practice

- What type of practice is it? Collaborative practice, partnership, or solo practice?
- How are the daily workflows in the practice? How busy is the practice?
- What is the staff composition and their roles? How are work tasks distributed and negotiated? What is the relationship between doctors and staff?
- How is the practice organized? Describe the general atmosphere and the waiting room environment.
- How do doctors perceive patients with severe mental illness, mild mental illness, and substance abuse? Do they perceive these patients as different from patients without mental illnesses?

## 2) Recruitment and the patients included in the project

- Are the patients being recruited according to the study criteria, including diagnoses, severity, age, etc.?
- How many patients are recruited per doctor, and what is the recruitment process like?
- Are the patients recruited for the study frequently visiting the practice already or are they “rare guests”?

## 3) Patient access to the practice

- How do practice personnel and the patient agree about the proper consultation time? Who in the practice makes the initial contact - secretary, nurse, doctor, or others? Is there an existing relationship with the patient?
- How does the practice ensure that patients feel welcome and accommodated? How is waiting time managed in the waiting room?
- Prior to the consultation, how long has the patient been with the practice? How frequently has the patient visited the general practice within the last 2 years?

## 4) Introduction to the study and data collection

- How did the doctors experience the initial introduction to the study? Did it meet their needs? What was missing? How did they communicate the study to their staff?
- How are the pull lists made for data extraction? What potential problems arise, and what software is used for extraction?
- Who is excluded from the lists, and why? Who is included, and what are the reasons for their inclusion?
- How many patients are recruited per doctor, and how is the recruitment process conducted?
- What recruitment methods have been found effective? How do patients experience the recruitment process?
- How do doctors and staff work to make patients feel welcome in the study?
- How does the study's implementation (e.g., using SOFIA) impact everyday practice? Does it change the workload? How do the practice adapt to accommodate the study?
- Are there any specific needs or requirements regarding study materials or contact with the project office? How much support is necessary?
- How does data generation work, and is it feasible in terms of time consumption?
